# Stem Cell-Based Hair Cell Regeneration and Therapy in the Inner Ear

**DOI:** 10.1007/s12264-023-01130-w

**Published:** 2023-10-03

**Authors:** Jieyu Qi, Wenjuan Huang, Yicheng Lu, Xuehan Yang, Yinyi Zhou, Tian Chen, Xiaohan Wang, Yafeng Yu, Jia-Qiang Sun, Renjie Chai

**Affiliations:** 1 State Key Laboratory of Digital Medical Engineering, Department of Otolaryngology Head and Neck Surgery, Zhongda Hospital, School of Life Sciences and Technology, Advanced Institute for Life and Health, Jiangsu Province High-Tech Key Laboratory for Bio-Medical Research, Southeast University, Nanjing, 210096 China; 2 Hospital of Southeast University, Nanjing, 210096 China; 3 First Affiliated Hospital of Soochow University, Suzhou, 215006 China; 4 Department of Otolaryngology-Head and Neck Surgery, The First Affiliated Hospital of USTC, Division of Life Sciences and Medicine, University of Science and Technology of China, Hefei, 230001 China; 5 Department of Otolaryngology Head and Neck Surgery, Sichuan Provincial People’s Hospital, University of Electronic Science and Technology of China, Chengdu, 610072 China; 6 Co-Innovation Center of Neuroregeneration, Nantong University, Nantong, 226001 China; 7 Institute for Stem Cell and Regeneration, Chinese Academy of Science, Beijing, 100101 China

**Keywords:** Hearing loss, Cochlea, Stem cell, Hair cell regeneration

## Abstract

Hearing loss has become increasingly prevalent and causes considerable disability, thus gravely burdening the global economy. Irreversible loss of hair cells is a main cause of sensorineural hearing loss, and currently, the only relatively effective clinical treatments are limited to digital hearing equipment like cochlear implants and hearing aids, but these are of limited benefit in patients. It is therefore urgent to understand the mechanisms of damage repair in order to develop new neuroprotective strategies. At present, how to promote the regeneration of functional hair cells is a key scientific question in the field of hearing research. Multiple signaling pathways and transcriptional factors trigger the activation of hair cell progenitors and ensure the maturation of newborn hair cells, and in this article, we first review the principal mechanisms underlying hair cell reproduction. We then further discuss therapeutic strategies involving the co-regulation of multiple signaling pathways in order to induce effective functional hair cell regeneration after degeneration, and we summarize current achievements in hair cell regeneration. Lastly, we discuss potential future approaches, such as small molecule drugs and gene therapy, which might be applied for regenerating functional hair cells in the clinic.

## Introduction

Hair cells in the cochlea of the inner ear are responsible for the detection of sound vibration and for the transformation of these mechanical signals into electrical signals, which are subsequently transmitted by the spiral ganglion neurons (SGNs) to the brain [1]. The afferent auditory fibers from the SGNs terminate at the cochlear nucleus, which is where the first step of centralized auditory processing occurs. Fibers in the cochlear nucleus project to the contralateral superior olivary complex, and the inferior colliculus receives the input information from the superior olivary complex and processes and delivers the sound frequencies to the medial geniculate nucleus. Sound information is finally integrated into the primary auditory cortex and the secondary auditory cortex of the temporal lobe [2]. Damage to hair cells due to gene mutations or environmental factors such as noise exposure, ototoxic drugs, chronic infections, etc., causes mild to profound hearing loss. It is, therefore, crucial to understand the damage-related mechanisms of hair cell injury, the regulation of hair cell development, and potential methods for hair cell regeneration. Research into anatomy and developmental biology has helped researchers discover and verify the structures and functions of hair cells and has led to novel approaches to regenerating new hair cells from neonatal or adult stem cells and precursor cells.

Hair cell development and regeneration are regulated by multiple signaling pathways that interact with each other to form a signaling network [3]. Stem cell technologies have been used to establish a number of inner ear models for studying hair cell regeneration. A variety of hair cell regeneration models derived from stem cells have been developed, including embryonic stem cells (ESCs), pluripotent stem cells, and inner ear stem cells [4–6]. Currently, drug therapy based on small molecules and gene therapy mediated by adeno-associated virus (AAV) have been developed and have achieved some degree of hair cell regeneration. To further improve the results of these therapies, it is important to understand the mechanism of hair cell regeneration and the properties of the new hair cells that are derived from different stem cells. In this review, we introduce the field of stem cell regenerative medicine in hair cell regeneration and summarize the latest progress and prospects of hair cell regeneration therapy.

## Structure and Function of the Cochlea

The inner ear consists of two main parts, the cochlea, and the vestibule, that are responsible for sound transduction and balance, respectively. As a sensory organ of the auditory system, the cochlea transmits sound to the brain through the auditory nervous system [7]. The cochlea is a spiral-shaped bony labyrinth with a length of about two and a half turns along the central cochlear axis and is referred to as the modiolus in humans. The spiral canal of the cochlea is divided into the scala vestibule, scala tympani, and scala media by its internal basilar membrane and Reissner's membrane.

**Fig. 1:**
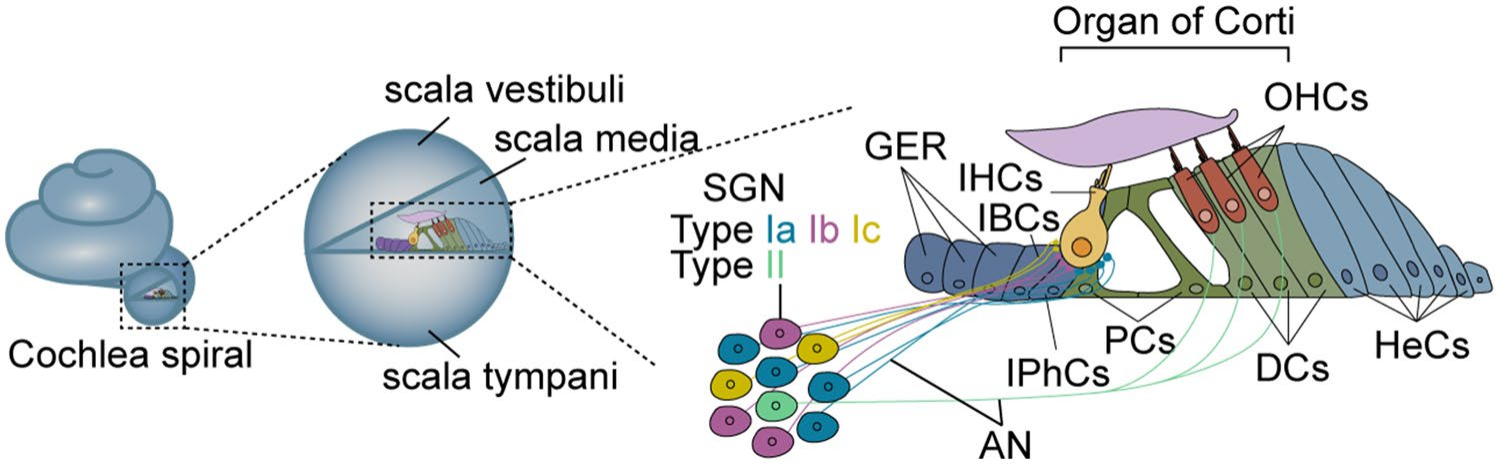
Structure of the cochlea. The cochlear duct is a spiral-shaped bony labyrinth with three separate cavities called the scala vestibule, scala media, and scala tympani. The sensory epithelium includes three rows of OHCs, one row of IHCs, and supporting cells (GER cells, inner border cells (IBCs), inner phalangeal cells (IPhCs), pillar cells (PCs), Deiters’ cells (DCs), and Hensen’s cells (HeCs)) and SGNs. AN: auditory nerve

There are three kinds of functional cell types in the cochlea – namely hair cells, supporting cells, and SGNs – and there is a single row of inner hair cells (IHCs) near the modiolus and three rows of outer hair cells (OHCs) located away from the cochlear modiolus. The perception of sound relies on the conversion of mechanical stimuli into bioelectrical signals by the cochlear hair cells. The signals are further transmitted to the afferent neurons in the spiral ganglion. OHCs are responsible for amplifying sound vibrations, and stereocilia, which are special mechanosensitive organelles on the apical surface of hair cells, deflect in response to the amplified auditory stimuli. As a consequence, the tension on the tip-link is increased and mechanoelectrical transduction channels open on the tip of the stereocilia resulting in hair cell depolarization and rapid neurotransmitter release from the ribbon synapses at the base of the hair cells [1]. And, studies have shown that the ribbon-type synapses show great heterogeneities at the basolateral side of the IHCs [8]. The mammalian cochlea also contains several types of supporting cells. For example, Deiters’ cells and pillar cells provide mechanical support for the hair cells, and these cells, together with the supporting cells from the greater epithelial ridge (GER) form the resource pool of hair cell progenitors (Fig. 1) [3].

There are two types of SGNs in mammals – type I and type II – based on differences in their peripheral nerve innervation (Fig. 1). Type I SGN accounts for 90%–95% of all SGNs, and these are afferent nerves that are covered by a myelin sheath and that mainly innervate the IHCs and transmit information to the nucleus. Type II SGNs account for only 5%–10% of all SGNs and are unmyelinated afferent nerves that mainly innervate OHCs. Type I SGNs have variable spontaneous rates (SRs), and Liberman *et al.* characterized SGNs as having high SR (>18 spikes/s), medium SR (0.5–18 spikes/s), and low SR (<0.5 spikes/s) [9]. In 2018, Sun *et al.* and Shrestha *et al.* simultaneously demonstrated that type I SGNs come in three subtypes (Ia, Ib, Ic) [10, 11], and Shrestha *et al.* used sparse labeling of SGNs to show that the Ia, Ib, and Ic subtypes have matched the characteristics of the high, middle, and low SR subgroups, respectively [10].

## Signaling Pathways and Transcription Factors in Hair Cell Regeneration

**Fig. 2:**
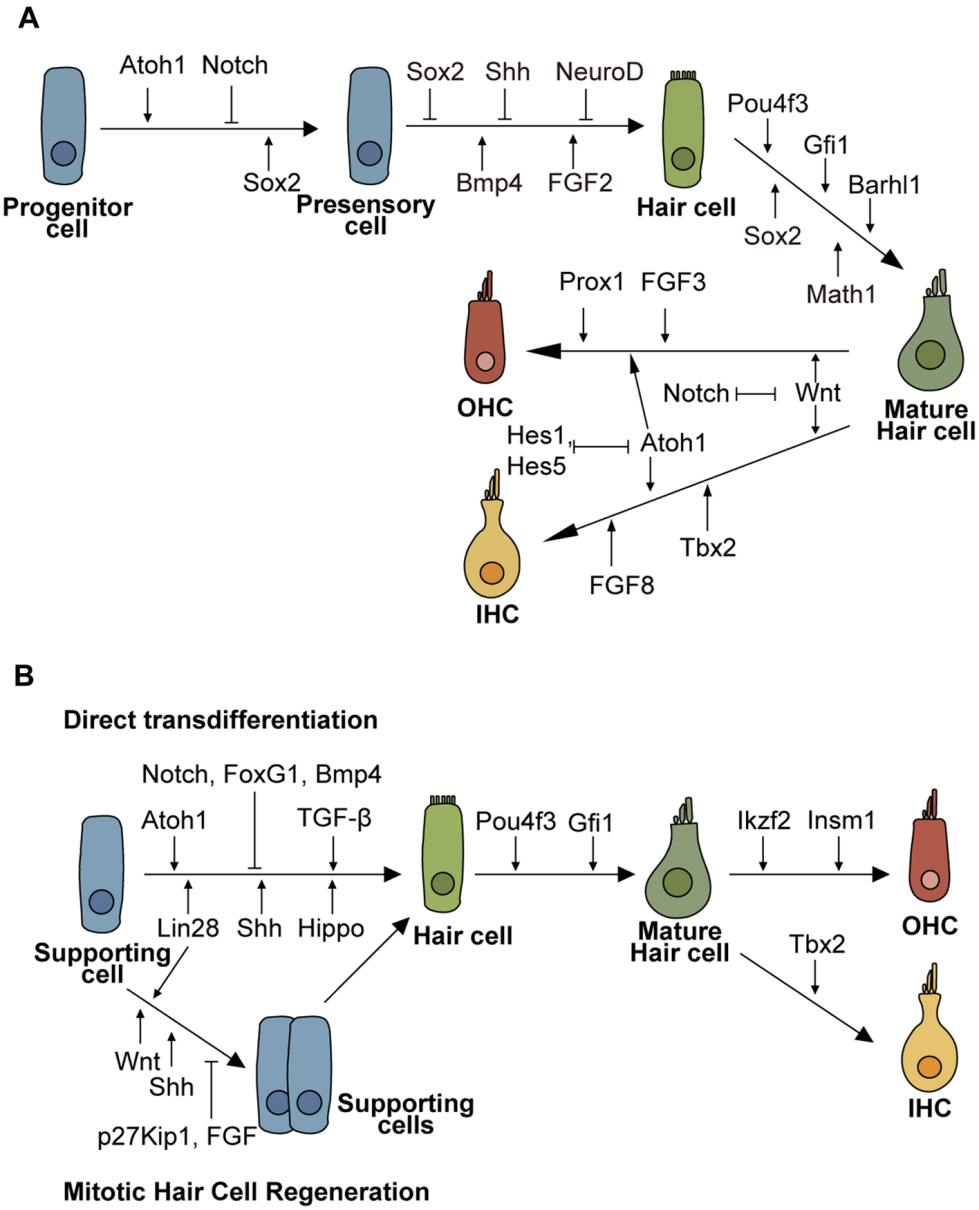
Signaling pathways and transcription factors involved in hair cell reprogramming. **A** Schematic of the hair cell developmental cascade. **B** The two pathways of direct hair cell transdifferentiation and mitotic hair cell regeneration in which supporting cells first undergo mitosis before differentiating into hair cells

Hair cells can be damaged through genetic mutations or as the result of continuous mechanical and chemical forces, including noise, aging, and ototoxic drugs. Recent studies have revealed some mechanisms behind hair cell damage, including autophagy, oxidative stress, apoptosis, tip link breakage, and synapse loss [12]. Hair cells do not spontaneously regenerate in vertebrates after the neonatal period, and thus hearing loss is generally not reversible. In contrast, studies on hair cell death in chicks induced by noise have shown the regeneration of sensory epithelial cells [13, 14]. Hair cells and supporting cells have a close relationship, and they come from a common pool of progenitor cells called prosensory cells [15, 16]. In recent decades, many important genes involved in regulating the development of hair cells have been discovered. For example, the transcription factor *Sox2* is essential for the establishment and maintenance of prosensory cells [17]. In addition, Atoh1 plays a key role in the induction of hair cells during inner ear formation [18, 19] and is a necessary transcriptional activator in the formation of inner ear hair cells [20]. Its reactivation in supporting cells is an important step in hair cell regeneration [21, 22]. Also, a variety of signaling pathways, including Wnt and Notch, have been found to be involved in regulating hair cell development and regeneration by controlling the expression of various transcription factors (Fig. 2).

### Key Transcriptional Factors

*Atoh1*, also known as *Math1*, is an essential helix-loop-helix transcription factor involved in hair cell differentiation, and its activity is mediated by *Hes1* and *Hes5*, which are downstream genes of the Notch signaling pathway. The inhibition of *Hes1* and *Hes5* activates the expression of *Atoh1*, thus promoting the differentiation of supporting cells into hair cells [23]. In the absence of Atoh1, cochlear and vestibular hair cells fail to generate during embryonic development [20]. The failure of hair cell genesis leads to the death of supporting cells in the cochlear sensory epithelium [24]. The overexpression of *Atoh1* can induce the ectopic regeneration of hair cells in the sensory and non-sensory regions [25, 26], and the new hair cells possess a similar morphology as native hair cells and have rudimentary mechano-transduction properties [27]. The regenerative efficiency of *Atoh1* decreases with age [27], and expression of *Atoh1* alone is not sufficient to regenerate mature hair cells with normal physiological functions. Thus, other synergistic transcription factors remain to be identified.

FOXG1 is a member of the Forkhead box (FOX) protein family and participates in the differentiation of the brain, inner ear, and other tissues and also plays an important regulatory role in mitochondrial energy metabolism and biosynthesis [28]. *FoxG1* is expressed widely in the cochlea, including the organ of Corti and the cells of the GER [29]. *FoxG1* is crucial for hair cell survival, and the lack of *FoxG1* results in the degeneration of hair cells. The specific knockout of *FoxG1* in hair cells increases the number of hair cells in the apical turn of the cochlea and in some parts of the middle turn at postnatal day 1 and day 7, and these extra hair cells gradually undergo apoptosis and show decreased numbers at P21 along with hearing loss [30]. Conditional knockout of *FoxG1* in supporting cells promotes the direct transdifferentiation of supporting cells into hair cells through the downregulation of the Notch signaling pathway [31]. Despite these promising findings, it remains to be determined whether these regenerated hair cells have normal physiological functions.

Currently, three transcription factors involved in OHC and IHC fate determination have been identified. *Insm1* plays a role in critical stages of embryonic development and prevents OHCs from differentiating into IHCs [32], and deletion of *Insm1* leads to the expression of IHC-specific genes. *Ikzf2* is another key transcriptional regulator of OHC maturation [33], and lack of *Ikzf2* in OHCs leads to the reduction of critical OHC-expressed genes while ectopic expression of *Ikzf2* in IHCs up-regulates the expression of OHC-specific genes [33]. *Tbx2* is a key transcriptional regulator in IHC fate determination and maturation [34, 35], and knockout of *Tbx2* during embryonic development leads to the early expression of the OHC marker *Insm1*, ultimately leading to differentiation into OHCs [34].

Although these studies illustrate the promising potential of using a single signaling molecule such as Atoh1 as a sole agent for inducing hair cell fate in the differentiated cochlear epithelium, the newly-formed hair cells are very different from native hair cells in terms of both microstructures and functions. These transcription factors and the signaling molecules described in the next section have been considered to be the basis for the multi-gene co-regulation needed to regulate hair cell differentiation.

### Wnt Signaling Pathway

The Wnt signaling pathway is highly conserved in animals [36, 37], and the canonical Wnt/β-catenin signaling pathway is one of the main targets for mediating the regeneration of inner ear hair cells. Wnt/β-catenin is involved in the differentiation of auditory vesicles and auditory plaques in the early stages of mammalian inner ear development [38], and it is also involved in regulating the development of the cochlear canal and hair cell polarity [36, 39]. The target gene of the Wnt pathway is mainly located in the dorsal side of the auditory vesicles, and suppression of Wnt during mouse embryonic development results in the failure of the semicircular canal to form properly [40]. The Wnt targets *Lgr5*, *Frizzled9*, and *Axin2* are expressed in neonatal cochlear progenitor cells [41–43], and enhancement of canonical Wnt signaling by Wnt agonists facilitates the proliferation of *Lgr5*+ progenitors and the subsequent differentiation of hair cells [44]. The combined expression of *Atoh1* and *β-catenin* in cochlear progenitors leads to a ten-fold increase in hair cell regeneration [45]. In addition, activation of the Wnt signaling pathway can prevent hair cell necrosis and shedding caused by ototoxic drugs [46]. Wnt signaling encourages hair cell differentiation and prevents chemical-induced hair cell death, but it cannot stimulate hair cells to engage in physiological processes, thus preventing them from regenerating in a functional manner.

### Notch Signaling Pathway

The Notch signaling pathway in mammals is composed of Notch receptors, Notch ligands (DSL proteins), and intracellular effector molecules, and the Notch signaling pathway is indispensable for the differentiation of supporting cells and hair cells during the development of the inner ear [47]. Notch has several different ligands, and each ligand plays a different role in the development of the inner ear. *Jagged1* interacts with Notch to promote the formation of cochlear sensory precursor cells, and its absence leads to the loss of inner ear sensory epithelium [48]. Conversely, the knockout of *Jagged2* significantly increases the number of hair cells [49, 50]. *Jagged2* activates the expression of *Jagged1* in adjacent cells through a positive feedback mechanism and further initiates the differentiation of hair cells [51]. The activation of Notch signaling results in the reduced expression of Notch ligand and reduced *Atoh1* signaling in cells and thus the differentiation of supporting cells, while neighboring cells differentiate into hair cells [27]. Targeted knockout or down-regulation of Notch signaling promotes hair cell formation by expressing and accumulating *Atoh1* in supporting cells [50, 52].

Inhibition of Notch activity promotes the transformation of supporting cells into hair cells through both mitotic and non-mitotic mechanisms [47, 53]. Mitotic supporting cells can be detected in the Sox2^CreER/T2^-Notch1^flox/flox^ cochlea with a slight increase in the number of supporting cells, while a dramatic increase in the number of hair cells is seen in the OHC region [53]. Thus, Notch inhibition induces hair cell regeneration with the concomitant loss of supporting cells. The loss of supporting cells in turn leads to the death of hair cells, including the newly differentiated hair cells [54]. Therefore, Notch inhibition alone is not an ideal approach for long-term hair cell regeneration. The best way to promote hair cell regeneration is to first stimulate the proliferation of supporting cells and then differentiate the proliferating supporting cells into hair cells.

### Hedgehog Signaling Pathway

The transcription products of Hedgehogs regulate gene expression in distal and proximal cells, and this activity is critical for the morphogenesis of the dorsal-ventral axis of the inner ear. Hedgehog signaling also participates in the formation of the pro-sensory region [55] and in the development of progenitor cells and hair cells during embryonic inner ear development [56]. The sonic hedgehog (Shh) pathway interacts with the Wnt pathway in the development of the inner ear and induces the expression of *Atoh1* in hair cell progenitors [40, 57]. Knockout of key genes in the Shh pathway has a negative impact on ventral polarity in the development of the inner ear, leading to structural abnormalities and developmental disorders of the inner ear [58]. The activation of Shh signaling promotes supporting cell proliferation and hair cell regeneration in the cultured postnatal cochlea after neomycin exposure [59, 60], and recombinant Shh protein effectively promotes cochlear organoid formation and hair cell differentiation from *Lgr5*+ progenitors in the neonatal mouse cochlea [59]. A correlation between Shh activity and the differentiation of hair cells has been observed, but the underlying mechanisms of Shh in hair cell genesis remain unclear and follow-up studies need to be carried out.

### Hippo-YAP Signaling Pathway

The Hippo-YAP signaling pathway includes MST1/2, SAV1, LATS1/2, etc. The signaling stimulations, like growth factors, activate MST1/2 and start a cascade of downstream reactions. After LATS1/2 is activated, YAP can be phosphorylated, leading to the ubiquitination-based degradation of YAP in the cytoplasm [61, 62]. In the absence of the phosphorylation signal, YAP is transported into the nucleus where it activates gene transcription. YAP is widely expressed in mouse cochlear hair cells and supporting cells [63], and YAP inhibition by small molecules effectively promotes the initial stages of mitotic hair cell regeneration [64]. *Lats1/2* deletion or Lats-mediated inhibitory phosphorylation can overcome the proliferative quiescence in utricular supporting cells [63], while the loss of MST1/2 activity in the neonatal cochlea promotes non-mitotic hair cell generation synergistically with the Notch pathway [65].

### FGF Signaling Pathway

Fibroblast growth factors (FGFs) are encoded by the *Fgf* gene family and are involved in embryonic growth and development as well as homeostasis in adult organisms. During the development of the inner ear, the FGF signaling pathway is involved in the induction of the otic placode, the formation of the spiral ganglia, and the development of the sensory epithelium of the inner ear [66–69]. After treatment with ototoxic drugs, the supporting cells in the cochlea undergo massive proliferation due to the down-regulation of FGF signaling. FGF has an anti-proliferation effect on supporting cells, and the decrease in FGF triggers cell cycle re-entry [70]. In addition, blocking FGF significantly inhibits hair cell regeneration in the lateral line organs of zebrafish [71]. Studies have shown that FGF3 and FGF10 can induce the expression of early marker genes in inner ear development in cultured human ESCs, such as *Six1*, *Pax2*, *Pax8*, etc. [72, 73]. bFGF alone is used to induce human pluripotent stem cells to differentiate into otic placode cells, but with very low efficiency [74], suggesting that the differentiation process requires the cooperation of multiple signaling pathways [71]. In addition, the spatiotemporal expression of the FGF signaling pathway plays an important role in hair cell differentiation. Thus, optimal induction efficiency might be achieved by constantly adjusting the spatiotemporal order of gene expression.

### LIN28/Let7

LIN28 is an evolutionarily conserved RNA-binding protein that is highly expressed in ESCs and is involved in self-renewal of ESCs [75, 76]. In mammals, there are two LIN28 family members, LIN28A and LIN28B, that have been shown to be involved in the plasticity of supporting cells as well as hair cell differentiation and regeneration [77–79]. Yap is activated in damaged zebrafish lateral lines, and this leads to the upregulation of LIN28A expression in hair cell precursors, and the Wnt pathway acts as the downstream target of the LIN28A/*let7* axis to promote hair cell regeneration [77]. During cochlear development, LIN28B is expressed in undifferentiated prosensory cells and *let-7* is highly expressed in differentiated hair cells and supporting cells. Expression of LIN28B but not *Let7* in early embryonic prosensory cells leads to cell cycle withdrawal and differentiation defects in both hair cells and supporting cells. In late embryonic development, prolonged LIN28 and *let-7* downregulation in the absence of Notch signaling increases the direct conversion of supporting cells to hair cells [78]. Moreover, LIN28B overexpression overcomes the obstacle of regenerative capacity during supporting cell maturation by increasing Akt-mTORC1 activity [79].

Despite the important roles of these genes in hair cell differentiation and development, the effect of their long-term regulation on the development and survival of new hair cells remains unclear. For example, the timely withdrawal of *Atoh1* activity after hair cell genesis is critical for maintaining postnatal hair cell survival, and we previously found that long-term expression of Atoh1 in hair cells can cause severe injury [80]. Therefore, it is important to identify the mechanisms and regulatory factors as well as the timing of their activity at key time points during hair cell development in order to develop novel gene therapy methods.

## Multi-gene Co-regulation in Hair Cell Reprogramming

The classical signaling pathways and transcriptional factors in hair cell regeneration such as Wnt, Notch, Atoh1, Hippo/Yap, etc., that have been described above are all involved in an intricate network of crosstalk with each other. Wnt activation and Notch inhibition strongly promote mitotic hair cell regeneration in the postnatal murine cochlea [53, 81]. Also, Notch inhibition together with Hippo suppression promotes hair cell regeneration, particularly through the direct transformation of supporting cells into hair cells [65]. The co-activation of Wnt and *Atoh1* in neonatal *Lgr5*+ progenitor cells improves the efficiency of hair cell generation [45], and Notch inhibition and the activation of Wnt and *Atoh1* in *Sox2*+ supporting cells induce ectopic hair cells in both the sensory and non-sensory-regions-of-the-cochlea [53]. Finally, Hippo/Yap signaling is involved in hair cell damage repair, and loss of *Mst1/2* activates the Hippo effector YAP1 and induces hair cell regeneration [65]. However, the newly generated hair cells remain immature and non-functional, and they do not express the terminal differentiation markers of OHCs and IHC such as PRESTIN or VGLUT3 nor do they acquire the morphological properties of mature hair cells.

Hair cell morphogenesis and maturation are regulated both spatially and temporally by a variety of signaling and transcription factors. Atoh1 determines the fate and development of hair cells by regulating Pou4f3, which is a hair cell-specific transcription factor that regulates the development and maturation of late embryonic hair cells [82]. Gfi1 is a target of Pou4f3 [83] and is required for hair cell differentiation and survival [2]. In 2019, Chen *et al.* regenerated mature and functional OHCs and IHCs by co-expressing Gfi1, Pou4f3, and Atoh1 (GPA) in postnatal cochlear supporting cells. The newly regenerated hair cells expressed the specific markers MYO7A, VGLUT3, and PRESTIN and were innervated by the auditory afferent neurons. Patch-clamp results showed that the new IHCs mimicked the development process of intrinsic cochlear hair cells [84]. In 2022, Iyer *et al.* found that pillar cells and Deiters’ cells do not respond to forced GPA expression [27], and the potential reason for this might be the different expression levels of reprogramming factors mediated by *Fgfr3-iCre* and *Sox9-iCre*. Notably, GPA enhances hair cell reprogramming ability in 8-day-old supporting cells. However, GPA only increases the number of hair cells and there is no change in the transcriptional profile [27]. Thus, there are still barriers to be overcome in order to induce hair cell maturation.

During the maturation and development of the neonatal mouse cochlea after birth, the ability of supporting cells to regenerate hair cells is greatly reduced. The combination of *p27Kip1* deletion with ectopic *Atoh1* expression overcomes the age-related decline in supporting cell plasticity, resulting in the transformation of supporting cells into hair cells in adult mice suffering from noise-induced damage. Deletion of *p27Kip1* upregulates Gata3, a co-factor of Atoh1 that is lost from supporting cells as they age. Gata3 and Pou4f3 promote Atoh1-mediated hair cell regeneration in the mature cochlea, and the activation of Pou4f3 alone, Gata3 and Atoh1 together, and Pou4f3 and Atoh1 together promote hair cell reprogramming in adult mice [85]. *Ikzf2* is specifically expressed in OHCs, and its functional mutation leads to developmental defects in OHCs [33], and the co-overexpression of *Atoh1* and *Ikzf2* in adult supporting cells induces OHC regeneration in P30 mice. The transcriptomes of new OHCs are closest to those of P1 wild-type OHCs [86]. Similarly, transient *Myc* and Notch activation enable supporting cells in adult mice to respond to exogenous Atoh1, which induces supporting cells to transform into hair cells [87]. In addition, transient co-activation of LIN28b and the activin antagonist follistatin enhances the regeneration ability of mature supporting cells through the up-regulation of the TGF-β signaling pathway [88]. Both transcriptomic and epigenetic analyses of cochlear hair cells at different ages show the epigenetic reduction of hair cell gene loci in the supporting cells of the mouse cochlea over the course of postnatal development [27], suggesting that in order to produce functional hair cells additional hair cell-specific transcription factors and epigenetic regulators are required to inhibit the expression of supporting cell-specific genes during reprogramming into functional hair cells.

## Stem Cells for Hair Cell Regeneration

**Fig. 3:**
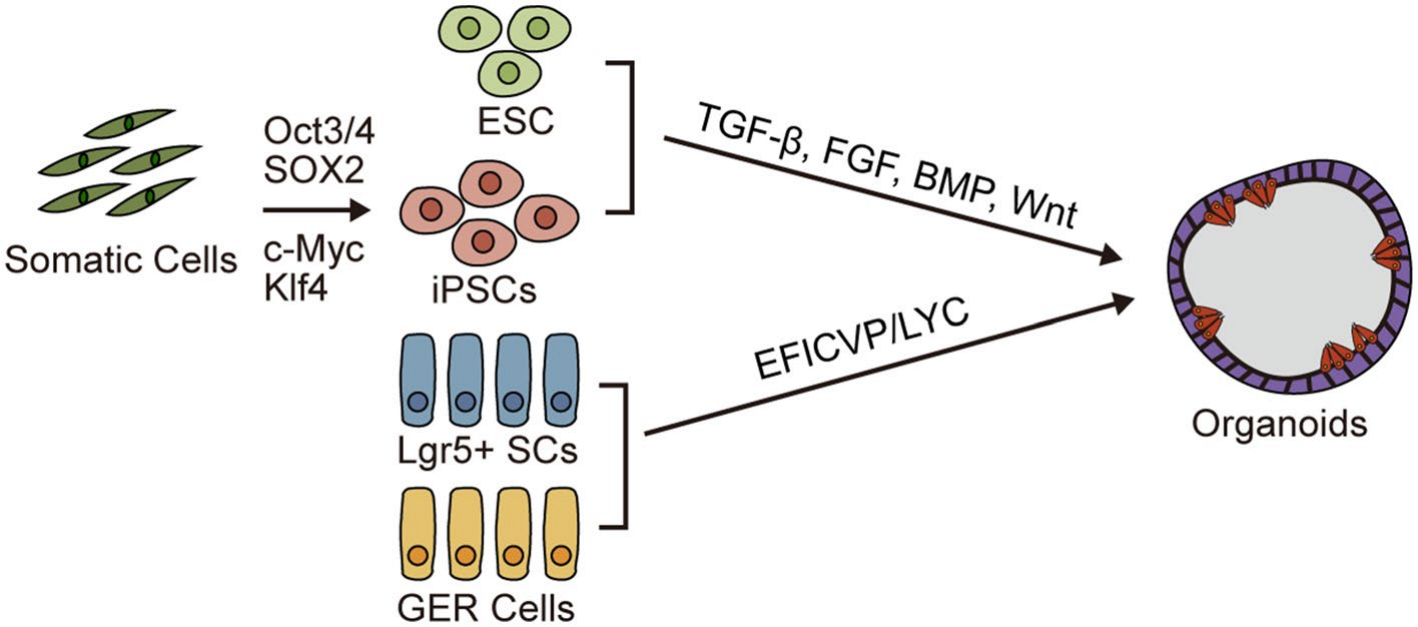
Stem cell-derived inner ear organoids. Four cell resources for cochlear organoid formation. The induction molecules are shown, respectively

Generally speaking, there are two models for studying hair cell regeneration in mammals, namely cochlear organoids and cochlear organs. Lateral line organs in Zebrafish, have been used in large-scale screenings looking for modulators of hair cell toxicity, protection, and regeneration [89]. Hair cells in zebrafish can spontaneously regenerate [90], which is completely different from the case of mammals, and thus they have limited application in studies of hair cell regeneration in mammals. Recently, organoids derived from pluripotent stem cells (PSCs) or from cochlear progenitor cells from neonatal mice have also been developed [4–6]. These organoids mimic the development of the inner ear, and the hair cells within the organoids share similar structural and functional properties as native hair cells [91–93]. The inner ear and cochlear organoids can be derived from ESCs, induced PSCs (iPSCs), *Lgr5*+ supporting cells, and GER cells (Fig. 3).

### Pluripotent Stem Cells

ESCs make up the foundation for mammalian development and have the potential to differentiate into any type of cell in an organism. Koehler *et al.* cultivated mouse ESCs in a three-dimensional system and were able to induce the differentiation of the inner ear sensory cell epithelium and reproduce the development process of hair cells [94]. Liu *et al.* used mouse ESCs for inner ear induction and developed organoids that morphologically resembled the vestibular organ of the inner ear, and the generated hair cells also had similar expression patterns of ion channels compared to native vestibular hair cells [95]. Also, the induced hair cells developed hair bundles and had comparable functions as type II vestibular hair cells. Co-culture is also a way to induce cell differentiation, and Carpena *et al.* co-cultured mESCs with the HEI‑OC1 hair cell line for 14 days and obtained both inner ear progenitor cells and hair cells [96].

iPSCs have similar characteristics as ESCs and avoid the ethical problems of using ESCs. In 2006, Takahashi and Yamanaka first proposed and demonstrated that the introduction of four transcription factors - *Oct3/4*, *SOX2*, *c-Myc*, and *Klf4* - can induce the trans-differentiation of mouse fibroblasts into PSCs [97]. Oshima *et al.* obtained inner ear progenitor cells from ESCs and iPSCs through directional induction, and these gradually acquired hair cell-like traits through the regulation of growth factors [98]. However, chromosomal aberrations that accumulate in PSCs during culture limit the expansion and differentiation of organoids [99]. Koehler *et al.* successively regulated TGF, BMP, FGF, and Wnt signaling, and this led to the induction of multiple vesicle-like structures in a single stem cell assembly [100]. At present, most methods for deriving inner ear organoids from PSCs can be improved using Koehler's stepwise induction, and the induced organoids are of the vestibular type and the hair cell-like cells look like type II vestibular hair cells [101]. Nie *et al.* proposed a method for developing inner ear sensory structures using hair cells from human PSCs, including human ESCs and iPSCs, through the modulation of BMP, FGF, and WNT using recombinant proteins and small molecules in a stepwise manner [5]. Tang *et al.* demonstrated the feasibility of creating organoids from disease models through the genetic manipulation of iPSC-derived organoids from deaf mice with the Tmprss3^Y260X^ mutation [102]. In addition, Menendez *et al.* directly transformed mouse embryonic fibroblasts, adult rat fibroblasts, and postnatal supporting cells using a combination of four transcription factors - *Six1*, *Atoh1*, *Pou4f3*, and *Gfi1* - and were able to successfully induce hair cell-like cells [103].

There are no substantial differences between ESCs and iPSCs in terms of their ability to differentiate along the inner ear lineage or in the functions acquired by the differentiated hair cell-like cells [104]. The electrical current responses obtained from mechanically stimulated ciliary bundles in regenerated hair cell-like cells are similar to those obtained from immature native hair cells, with small currents, broad current shift functions, highly variable presence and measured rates of adaptation, and no orientation sensitivity [100]. Morphological and electrophysiological analyses of regenerated hair cells suggest that a common signaling pathway triggers the development of mechanosensitive hair bundles and indicates the need for additional signals to specify hair cell subtypes, such as auditory or vestibular hair cells, inner or outer hair cells, and type I or type II hair cells [105].

Hair cell-like cells derived from ESCs or iPSCs using current techniques possess the morphological and electrophysiological characteristics of vestibular hair cells, but not cochlear hair cells. It is necessary therefore to identify the underlying mechanisms through which cochlear organoids are induced by ESCs or iPSCs, and the progressive events and critical time points for cochlear sensory epithelia development and directed hair cell differentiation need to be identified. Such knowledge will not only contribute to the construction of cochlear organoids but will also contribute to the functional differentiation of adult stem cells into hair cells.

### Inner Ear Stem Cells

Lgr5, a cell membrane receptor of the Wnt pathway, is an important marker of inner ear stem cells. Wang *et al.* showed that damage-activated *Lgr5*+ supporting cells can regenerate hair cell-like cells via proliferation and direct trans-differentiation both in cultured and native utricle tissue [106]. McLean *et al.* established a method to obtain large numbers of cochlear hair cells in organoids derived from isolated mouse *Lgr5*+ supporting cells, non-human primate epithelial cells, and healthy human inner ear tissue by pharmacological stimulation, including EGF, bFGF, IGF-1, CHIR, valproic acid (VPA), 2-phospho-L-ascorbic acid (pVc, a stable form of vitamin C) (EFICVP) and 616452 for organoid expansion and a combination of EFICVP, LY411575 (LY) and CHIR for hair cell differentiation [6]. Lenz *et al.* used the differentiated organoids derived from *Lgr5*+ supporting cells for drug screening, gene silencing, and overexpression, and for studying the genomic perturbations caused by CRISPR/Cas9 methodologies [107].

GER cells have the characteristics of prosensory cells, and Kubota *et al.* used single-cell RNA sequencing to determine different cochlear cell types in neonatal mice and to evaluate their potential to transform into inner ear organoids and identified three distinct groups of large epithelial crest cells with the ability to differentiate into inner ear organoids under high-density conditions [108]. Chen *et al.* used single-cell RNA sequencing to reveal the differentiation of eight cell subtypes from GER cells, two of which transdifferentiate predominantly into inner hair cells and two subtypes that ultimately differentiate into OHCs [109]. Iyer *et al.* found that some of the GER cells are not able to transform into hair cells, and this is partially due to the reconstitution of the Notch signaling interactions between newly-formed hair cells and GER cells [27].

Adult inner ear stem cells are an effective resource for in situ restorative regeneration of damaged hair cells, and inner ear stem cells are widely used in hair cell regeneration research. However, it is important to note that the stemness of the inner ear stem cells decreases rapidly after birth. Considering the clinical application, it is necessary to identify regulatory methods to completely restore the plasticity of stem cells in the inner ear.

## Hair Cell Regeneration Therapy

**Fig. 4:**
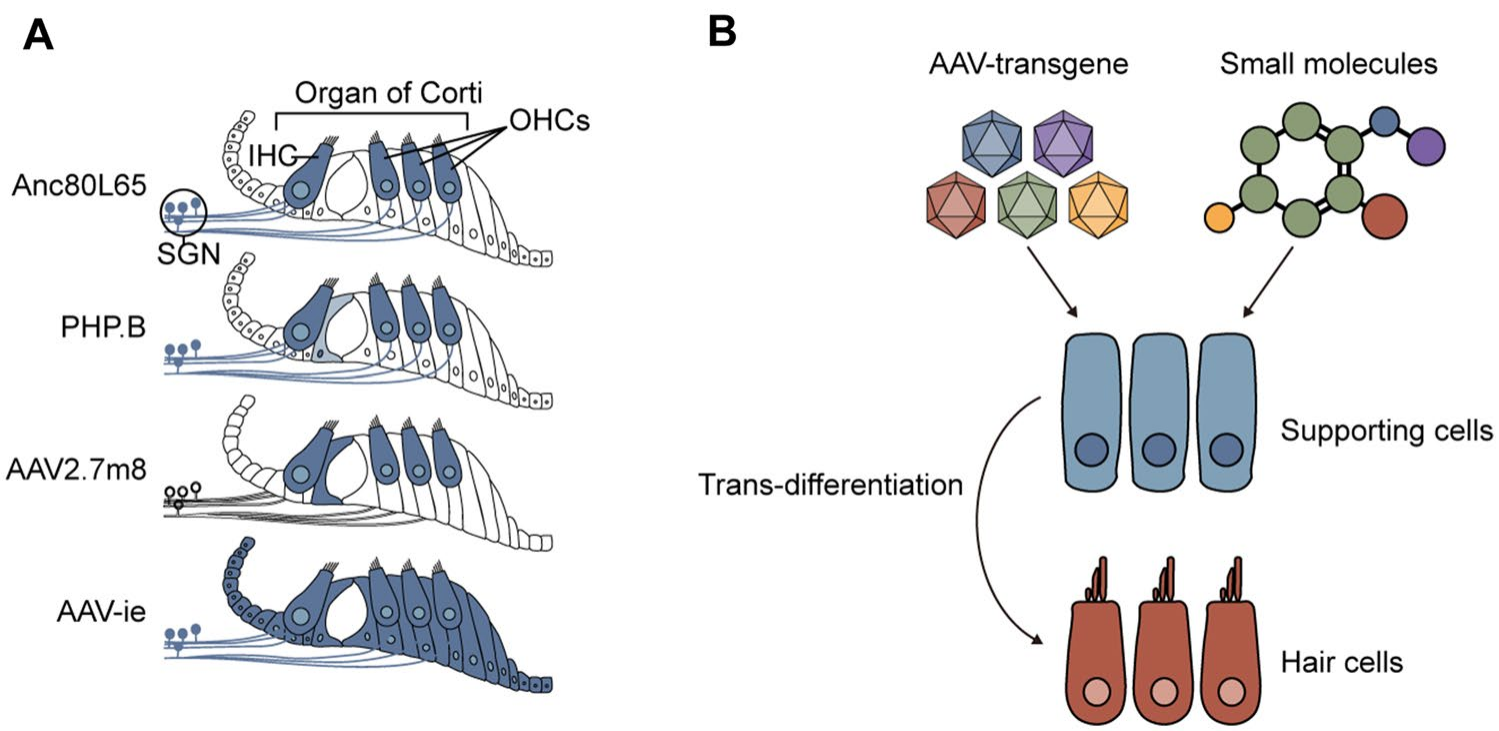
Hair cell regeneration therapy via AAV and small molecules. **A** Synthetic AAV vectors with high transduction rate in hair cells and supporting cells. The cells marked by dark blue indicate the cell types transduced by AAVs in the organ of Corti. The light blue indicates the low-efficiency selective expression of AAV-PHP.eGFP in the inner pillar cells driven by the *Gfap* promoter. **B** AAV-mediated transgene transduction, together with small molecules, facilitates multi-gene co-regulation during hair cell regeneration

Currently, inducing functional hair cells via inner ear stem cell differentiation to replace damaged hair cells is considered to be a feasible treatment for sensorineural hearing loss. The regulation of stem cell reprogramming is critical, and small molecule drugs and virus-mediated gene regulation are effective approaches (Fig. 4).

### Small Molecule Drugs

With the discovery of the important roles of several signaling pathways associated with hair cell regeneration in the inner ear, a variety of regulatory small molecule compounds have been screened and identified for hair cell regeneration.

Wnt signaling is necessary for hair cell differentiation in animals [36]. McLean *et al.* used a small-molecule cocktail to achieve a high yield of supporting cell proliferation and hair cell differentiation from *Lgr5*+ cells in cochlear organoids. They found that the glycogen synthase kinase 3β (GSK3β) inhibitors CHIR99021 and bFGF had the greatest effect on the number and percentage of Lgr5-GFP progenitors. CHIR 99021 is also critical for Lgr5 expression. The EGF and TGF-β receptor (ALK5) inhibitor 616452 promoted the most Lgr5 cell growth, while the histone deacetylase inhibitors VPA and pVc promoted the most Lgr5 expression. Only minimal effects of IGF-1 on Lgr5 cell number and percentage were detected. LY411575, a γ-secretase inhibitor for Notch inhibition, is usually used for hair cell differentiation together with CHIR 99021, not for organoid expansion [6]. Some of the molecules were successfully used to regenerate hair cells in organoids derived from adult rhesus macaque and human inner ear epithelia and from cultured cochlear explants [6].

In the cochlea, Wu *et al.* preserved *Lgr5*+ supporting cells and strongly promoted mitotic hair cell regeneration by simultaneously inhibiting Notch signaling using the γ-secretase inhibitor DAPT and activating Wnt signaling using the *β-catenin* nuclear translocation agonist QS11 [110]. Kastan *et al.* found that TRULI is a potent and non-toxic ATP-competitive inhibitor of *Lats1/2* in the Hippo signaling pathway and that it initiates the regeneration of mitotic hair cells [65].

Liu *et al.* optimized, characterized, and utilized the mouse cochlear organoid platform to perform high-throughput screening of more than 1,000 FDA-approved small molecule drugs and found that regorafenib, a tumor therapy drug, can significantly promote the differentiation of hair cells in cochlear organoids. Further studies showed that regorafenib also effectively promoted hair cell regeneration and maturation in cultured cochleae. This process is mediated by the VEGFR-MEK-TGFB1 signaling axis [111].

Frequency Therapeutics assessed the safety and audiometric effects of CHIR-911 and VPA, referred to as FX-322 in their study, to restore noise-induced or sudden sensorineural-related hearing loss by reprogramming progenitor cells to activate innate hair cell regeneration potential in humans. Intratympanic FX-322 dosing was performed in individuals with mild to moderately severe chronic sensorineural hearing loss. The primary measurement index of the study was speech recognition performance, which is a test of hearing loss based on the evaluation of sound clarity and speech comprehension. Clinical studies of a single dose of FX-322 in the subjects showed improved speech recognition performance by 18%–42% with clinical significance [112]. FX-322 is the first pharmacologic therapy to show statistically and clinically significant hearing improvement in clinical trials for the treatment of sensorineural hearing loss. However, the results from phase II clinical trials showed that the primary efficacy endpoint for improving speech perception was not achieved, and there was no statistically significant difference in the rate of improvement in speech perception at day 90 between patients who received FX-322 and those who received a placebo. CHIR-911 and VPA activate the Wnt signaling pathway to promote the plasticity of adult supporting cells, but the regulation of Wnt signaling alone cannot achieve functional hair cell regeneration for hearing restoration.

### Virus-Mediated Gene Manipulation

Another effective method of multi-gene co-regulation in inner ear stem cells is the inner ear administration of exogenous transgenes through the round window membrane, semicircular canal administration, or cochleostomy, using viral and non-viral vectors. The advantages of non-viral vectors are that they are easy to prepare, have a large gene-carrying capacity, and do not induce immune responses in the host. However, their fatal defect is their low transduction efficiency. Viral vectors are widely used due to their high transduction efficiency [113].

Clinically, viral vectors for gene therapy usually include recombinant AAV, retrovirus, lentivirus, etc. In 2005, Izumi *et al.* first transduced *Atoh1* into the inner ear of deaf guinea pigs via adenovirus (Ad) vectors. Ad-*Atoh1* achieved partial hearing recovery and improvement by inducing hair cell regeneration [114]. Similarly, Chen *et al.* delivered Ad-*Math1* combined with *Pax2* to promote hair cell regeneration after neomycin treatment in the neonatal mouse organ of Corti [115]. In 2012, Burns *et al.* used co-transduction of Ad-mediated *Oct3/4*, *Klf4*, *Sox2*, and *c-Myc* with the anti-degradation T58A mutants to trigger a significant response in terms of proliferative stimulus and acceleration of S-phase entry of supporting cells in the adult utricle [116]. In the adult cochlea, transient activation of *Myc* and Notch by Ad-mediated delivery enabled the transformation of supporting cells into hair cells in response to *Atoh1* administration [87]. Finally, Lu *et al.* used Ad-*YAP* to induce supernumerary hair cells after neomycin exposure in cultured cochleae [65]. Despite these promising findings, the potential immune and carcinogenic effects of Ads limit their application in the clinic.

Adeno-associated viruses are currently being used in numerous ongoing clinical trials listed on Clinicaltrials.gov. The natural AAV has been genetically modified in which the *rep* and *cap* genes have been replaced by therapeutic exogenous transgenes flanked by two ITR sequences to produce a vector suitable for the transgenic application. Several therapeutic AAV drugs have been approved by the European Commission or the U.S. Food and Drug Administration for use in patients, including Glybera (UniQure, AAV1-LPL), Luxturna (Spark Therapeutics, AAV2-RPE65), and Zolgensma (Novartis, AAV9-SMN1). Natural AAVs inefficiently transduce cochlear cells, but recently researchers have discovered some AAV vectors that can be transfected into inner ear stem cells. Landegger *et al.* produced an ancestral AAV2/Anc80L65 that could effectively target cochlear and utricular hair cells based on the in silico design of the lineage of the natural AAV serotypes 1, 2, 6, 8, and 9 [117]. PHP.B, a variant based on AAV-9, showed a similar transduction rate in OHCs and IHCs as Anc80L65 [118]. However, these serotypes have shown little improvement in the transduction of supporting cells. Subsequently, AAV2.7m8 was synthesized and was used to verify the preferential targeting of cochlear hair cells and some of *Lgr5*+ supporting cells, including inner pillar cells (80%~90%) and inner phalangeal cells (50%~70%) [119]. Tan *et al.* constructed a mutant AAV-inner ear (AAV-ie) vector based on AAV-DJ by inserting the polypeptide DGTLAVPFK, which improved the transduction rate in supporting cells (>80%) by generating transmembrane structures [120].

AAV-ie efficiently transduces cochlear supporting cells, hair cells, and vestibular hair cells, with improved rates in Deiters' cells (~80%) and inner phalangeal cells (~ 90%). AAV-ie has no effect on the cochlear epithelia or auditory system and is used in hair cell regeneration therapy [120–122]. A variant of AAV-ie, AAV-ie-K558R, has a similar effect on the transduction of supporting cells and hair cell regeneration [123]. However, the non-specificity of the developed AAVs for supporting cells limits their applications in functional hair cell regeneration. The development of highly effective and specific AAVs targeting supporting cells is critical for research into the regeneration of hair cells.

## Conclusions

The non-regenerative loss of hair cells is the main cause of deafness. A large number of studies have looked at ways of promoting the renewal and proliferation of hair cell progenitor cells for hair cell regeneration in order to rebuild the auditory organ. This is a promising treatment method for hearing loss, and how to regulate and activate inner ear stem cells to make them proliferate and differentiate and thus regenerate functional hair cells is the key to auditory injury repair. Atoh1, Wnt, Notch, and other complex signals interact to form a complex regulatory network for hair cell regeneration and maturation. However, functional hair cell regeneration remains a major challenge. An increased understanding of the fate regulation of inner ear stem cells for hair cell regeneration and maturation after damage and the exploitation of clinically applicable therapeutic methods will greatly accelerate the clinical use of stem cells for restoring hearing function.
